# Modeling Rainfall Variability over Urban Areas: A Case Study for Kuwait

**DOI:** 10.1100/2012/980738

**Published:** 2012-05-03

**Authors:** Jaber Almedeij

**Affiliations:** Civil Engineering Department, Kuwait University, P.O. Box 5969, Safat 13060, Kuwait

## Abstract

This study examines the spatial and temporal variability of monthly total rainfall data obtained from weather stations located in the urban areas of Kuwait. The rainfall data are analyzed by considering statistics on a seasonal basis and by means of periodogram technique to reveal the periods responsible for the variable pattern. The results demonstrate similarity implying that a point estimate of rainfall data can be considered spatially representative over the urban areas of Kuwait. A sinusoidal model triggering the influence of the detected periods is developed accordingly for the time duration from January 1965 to December 2009. The model is capable of describing the rainfall data with some discrepancies between the actual and calculated values resulting from hidden periods that have not been taken into account. This finding suggests that the ability to construct a more reliable model would require a wider range of historical data to detect the other periods affecting the rainfall pattern.

## 1. Introduction

The development of a model describing the rainfall pattern over a catchment has been a prime focus of hydrological research for many decades. Among the variety of applications, rainfall models have been used to develop storm hyetographs so that to simulate streamflow records [1], assess soil-moisture for crop water requirements [2], estimate water balance for reservoirs and groundwater aquifers [3], and analyze flood frequency to avoid the risk of road ponding especially at stormwater drainage inlets located at in-sag points [4, 5], as road ponding is considered a major contributor to traffic accidents and delays as well as nuisance and possible hazard to pedestrians [6].

 For Kuwait, the development of a rainfall model is relevant to establish an efficient rainwater harvesting system that has a potential to provide an additional source of water for agriculture and other applications [7], as the country facing a water shortage problem with only one conventional resource of fresh groundwater located in the north with limited quantities. Kuwait, which is about 18,000 km^2^, is a dessert country characterized by long, hot, and dry summer, and short winter. Temperature during summer (winter) reaches an average of 44°C (15°C) during the day time, with the lowest average falling to 23°C (3°C) during the night. The total amount of rainfall through the year varies between 30 and 250 mm, most of which falls between November and April due to western pressure depressions.

Owing to the high soil's infiltration properties of Kuwait, the collection of surface water runoff through a rainwater harvesting system would be much more efficient in the urban areas than in the desert [8]. The urban catchments of Kuwait, with a total area of about 600 km^2^, have a storm sewer system of separate type where the collected water is currently discharged at the outfalls into the sea. This existing drainage system could be used directly for harvesting, although the collected rainfall would need to be treated more thoroughly before it is used. Studying the economic feasibility of integrating a complete harvesting system for the urban catchments may be accomplished by the estimation of water input using a rainfall model.

A comprehensive analysis of rainfall variability of Kuwait was presented by Marcella and Eltahir [9] at both seasonal and interannual time scales. They concluded that a rainfall model can be developed for the country if the periodic pattern at both seasonal and interannual time scales has been understood. Despite the local weather stations available, the data obtained are not of sufficient quantity, both spatially and temporally, to carry out a comprehensive analysis of the rainfall pattern. The only historical data consistent is that produced from a weather station located in Kuwait International Airport. Marcella and Eltahir also added rainfall datasets from two other sources, from the Climate Research Unit and the Global Precipitation Climatology Project. Owing to the different techniques adopted to produce the datasets, discrepancies among the rainfall measurements have been noticed. However, the records of Kuwait International Airport consistently fall in between the two other datasets and may accordingly be considered here in an objective manner to provide a quantitative analysis.

 The aim here is to model the rainfall pattern over the urban areas of Kuwait. Data available from local weather stations will be employed to examine the spatial variability of rainfall within the urban areas. Then, the temporal rainfall variability will be studied using a more comprehensive data obtained from Kuwait International Airport. A possible model based on considering the spatial and temporal variability of rainfall will then be developed and discussed.

## 2. Rainfall Spatial Variability

**Figure 1:**
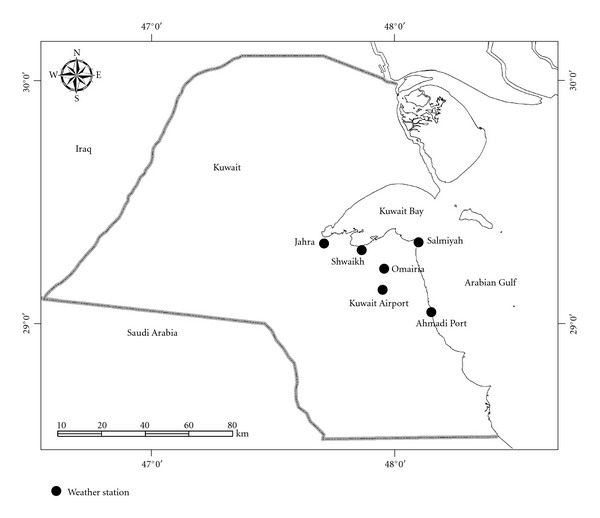
Weather stations in urban areas of Kuwait.

The spatial variability of rainfall can be examined over the urban catchments of Kuwait by employing monthly total rainfall data collected from the following weather stations: Jahra, Shwaikh, Salmiyah, Omairia, Kuwait International Airport, and Ahmadi (Figure 1). These stations are located in the urban areas within latitudes 29°20′ N and 29°03′ N, and longitudes 47°37′ E and 48°10′ E. As it was mentioned earlier, except that of Kuwait International Airport, the data collected from the weather stations are not of substantial continuity coverage. The only consistent data available for these stations are that within time duration from January 1994 to December 2005.

**Figure 2:**
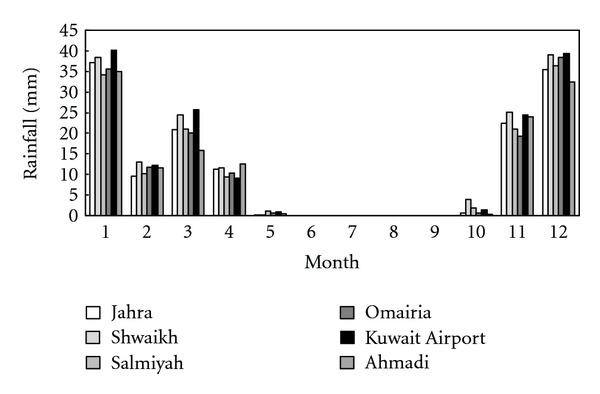
Seasonal mean of monthly total rainfall data calculated using (1) for the time duration from January 1994 to December 2005.

The average values of monthly total rainfall data can be measured by considering statistics on a seasonal basis using the expression

(1) $$\begin{array}{c} \overset{®}{P}=\frac{1}{N}\overset{N}{\underset{i=1}{\sum }}P_{i,\tau },\;\tau =1,\ldots ,12 \end{array},$$

where *P* is monthly total precipitation, defined as the total for month *τ* in the given year *i*; $\bar{P}$ is seasonal mean precipitation; and *N* is total number of years. The results are presented graphically in Figure 2. As can be seen, the data possess nearly equivalent seasonal means with small differences of ±3 mm. This finding is in agreement with the conclusion drawn by others [9] who found that the average values of rainfall data collected from these stations are not that different since they are sufficiently close in distance, while spatial distribution is evident within a larger scale.

The pattern of the monthly total rainfall can be compared for the different weather stations by plotting the periodogram, which is a Fourier transform of the autocovariance function representing an unsmoothed spectral plot for examining the cyclic structure in the frequency domain [10]. This technique is used to reduce the effect of the measurement noise and thus detect which frequencies within the range of time are most responsible for the data pattern. Typically, a large peak value shown in a periodogram corresponds to a period that is strongly represented in the time series. For example, a typical periodogram for monthly averaged temperature data can show a period of 12 months implying that 6 months of the year possess considerably lower temperatures than the other 6 months.

**Figure 3:**
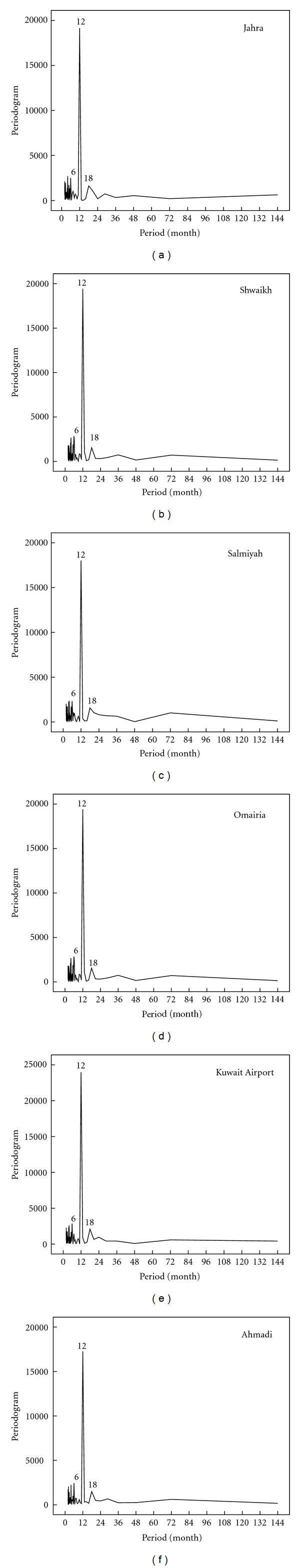
Periodograms for the monthly total rainfall data obtained from the weather stations in urban areas for the time duration from January 1994 to December 2005.

Figure 3 provides the periodograms of the rainfall data obtained from the weather stations. As can be seen, not only the seasonal means of the rainfall data are similar, but also the patterns. This is evident in the periodograms for the time duration between 6 and 24 months. This range of time has been considered here for the comparison since the minimum period of 6 months represents the distinct four seasons of the year, that is, spring, summer, autumn, and winter, by which smaller periods become difficult to justify using monthly data; while periods larger than about 24 months can be resolved more conveniently if a longer time series became available. Here, three periods are detected of 6, 12, and 18 months. The 12-month period is more pronounced than the others, with the power value (*y* axis) being about 10 times larger. Apparently, the 6- and 12-month periods are related to the seasonal variation of rainfall during the year typically observed in climatological data. The third period of 18 months indicates a variation in rainfall pattern of a longer time. The reason for the existence of this period in the data is unclear and may need further investigation to establish any relation.

## 3. Rainfall Temporal Variability

Owing to the similar seasonal mean and pattern, rainfall data collected for a point estimate can be considered spatially representative over the urban areas of Kuwait. The monthly total data of Kuwait International Airport can thus be considered representative and employed to examine the temporal variability of rainfall in a larger time scale. A full range of data is available with time duration from January 1965 to December 2009. It should be mentioned that some measurements have not been taken in this data from August 1990 to June 1991, corresponding to the Iraqi invasion of Kuwait. To maintain data continuity in terms of time, this lack of information has been handled here by averaging the data by considering the seasonal mean resulting from adding the value of the same month but for the year before and after and then dividing by two.

**Figure 4:**
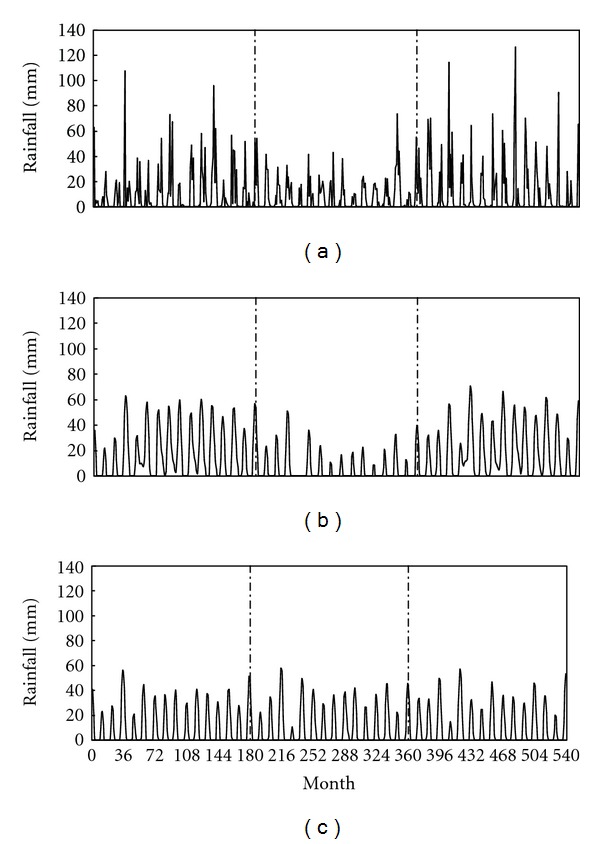
Monthly total rainfall for the weather station of Kuwait Airport for the time duration from January 1965 (corresponding to month number 0) to December 2009 (month number 540): (a) actual dataset; (b) calculated records based on periodic model; (c) calculated records based on periodic model with excluding period 360 months.

The monthly total data of Kuwait International Airport for the time from January 1965 to December 2009 are shown in Figure 4(a). Here, a distinct rainfall pattern can be noticed dividing the data into three time intervals of 180 months. The rainfall incidents for the first and third intervals are larger than that for the second. This is evident, given that the corresponding total rainfall amounts through the intervals are 1870, 1420, and 2160 mm, and the seasonal means estimated from (1) are 124.47, 94.93, and 144.25 mm. This pattern suggests the presence of a large period of 360 months that may not appear in the periodogram as its size is so large relative to the size of the data by which there would be no sufficient oscillating movement repetitive over the time interval.

**Figure 5:**
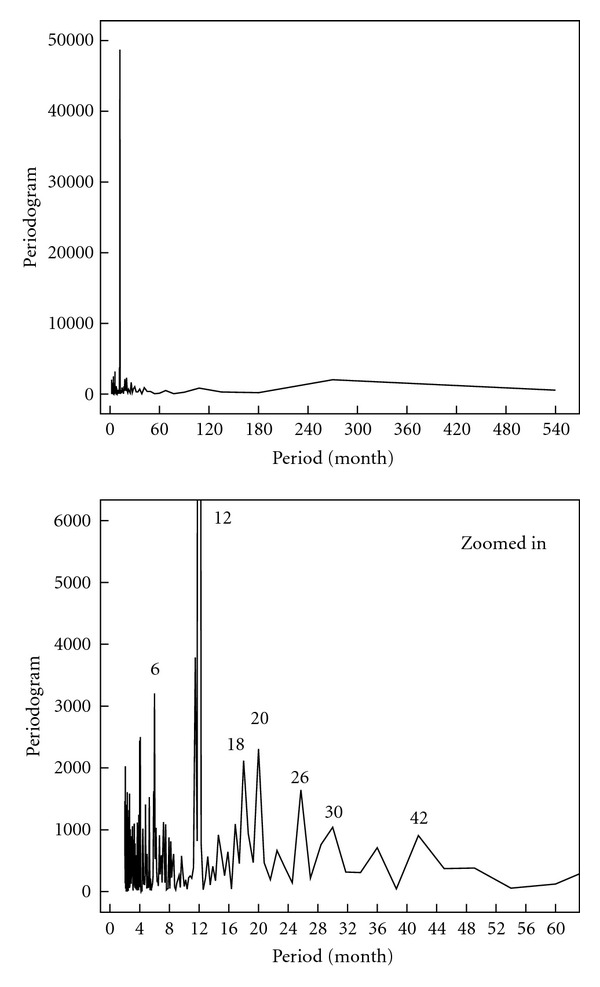
Periodogram for the monthly total rainfall data obtained from the weather station of Kuwait International Airport for the time duration from January 1965 to December 2009.

Figure 5 presents the periodogram of the data. If one neglects the peaks shown in the figure for the time less than 6 months, then the following periods dominate: 6, 12, 18, 20, 26, 30, and 42 months. Compared to the periodogram in Figure 3 for the same weather station, more periods appeared as the time scale of the data became longer. The periods of 6, 12, and 18 are similar to that found previously. The period of 20 months can be considered very close to that of the 18 months. The larger periods of 26 and 30 corresponding to patterns of nearly 2 and 2.5 years can either be related to solar activities [11] or to the well-known tropical Quasi-Biennial Oscillation (QBO), which is believed to be functioning as a conduit to transfer solar effects to lower altitudes. The QBO is a dominant natural oscillation in the equatorial lower stratosphere described as the phenomenon of reversal of wind directions; that is, for about one year the prevailing wind direction is easterly, while during the following year it is westerly [12, 13]. The period of 42 months could be either due to solar activities or harmonic correlation with the period of 20 months (i.e., 20 × 2 = 40). As it was expected, the period of 360 months cannot be identified in the periodogram since its power is not high enough. One may thus conclude that other periods could also exist, although they might not appear explicitly due to insufficient data.

## 4. Model Development

The data of Kuwait International Airport can be used to model the rainfall variability over the urban areas. Generally, a time series data can be represented by a decomposition model of additive type composed of deterministic and stochastic components. The deterministic component is described by trend and periodic parts, and it can be formulated in a manner that allows exact predictions. The trend describes a long movement of the variable lasting over the entire time of observation, while the periodic part describes oscillating movement repetitive over a specific time interval. The stochastic component of a time series data can never be estimated exactly as it is considered to be made of random effects. Although a stochastic component is taken to be sufficiently stationary in simple time series models, in most commonly considered situations it exhibits complicated statistical correlations.

**Figure 6:**
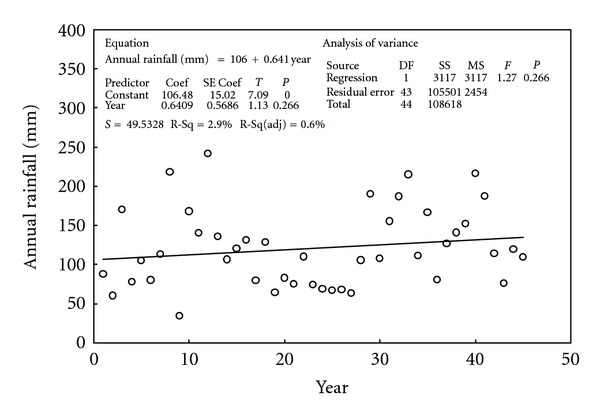
Annual total rainfall versus time for the data obtained from the weather station of Kuwait International Airport for the time duration from 1965 (corresponding to year number 1) to 2009 (year number 45). The solid line represents a trend fitted for the data using linear regression.

 While visual inspection of the monthly total data obtained from Kuwait International Airport suggests the presence of no trend but only periodic deterministic component, statistical inferences may be used to provide verification. This analysis can be accomplished using the annual total rainfall data. The claim to be tested is that a linear relation exists between the annual rainfall and time at a level of significance *α* = 0.05. As can be seen in Figure 6, the *P* value for the slope of the fitted linear relation is given as 0.266. This means that there is a 0.266 probability of obtaining a slope estimate as extreme as or more extreme than the one obtained if the null hypothesis of no linear relation was true. As the *P* value is more than the level of significance, the null hypothesis of no linear relation is accepted.

The periodic pattern of monthly rainfall data can now be estimated from the detected periods in the previous section. In general, a time-based data containing a periodic sinusoidal component with a known wavelength can be modeled using Fourier series, which can be expressed for multiperiods as

(2) $$s\left(t\right)=\overset{\infty }{\underset{n=1}{\sum }}\overset{k}{\underset{i=1}{\sum }}R_{n,i}\cos\left(2n\pi f_{i}t+\theta_{n,i}\right),$$

where

(3) $$\begin{array}{c} R_{n,i}=\sqrt{a_{n,i}^{2}+b_{n,i}^{2}},\\ \theta_{n,i}=\tan^{-1}\left(-\frac{b_{n,i}}{a_{n,i}}\right),\\ a_{n,i}=2f_{i}\overset{L_{i}+1/f_{i}}{\underset{L_{i}}{\int }}f\left(x\right)\cos\left(2n\pi f_{i}t\right)dx,\\ b_{n,i}=2f_{i}\overset{L_{i}+1/f_{i}}{\underset{L_{i}}{\int }}f\left(x\right)\sin\left(2n\pi f_{i}t\right)dx, \end{array}$$

where *s* is periodic sinusoidal component of rainfall; *R* is amplitude of variation; *f* is frequency, equal to the inverse of period; *θ* is phase angle; and *k* is total number of periodicities. The term (2*nπft* + *θ*) is measured in radians. As determined from the data, *k* value is equal to eight, and the values of *f* may be set by the periodic nature of rainfall data, that is, *f*_1_ = 1/6, *f*_2_ = 1/12,… cycles per month. The phase angle, *θ*, is necessary to adjust the model so that the cosine function crosses the mean, which is equal to zero for the data, at the appropriate time *t*. The difficulty is to determine analytically the function *f*(*x*) in Fourier coefficients. However, because of the existing randomness, a more simple procedure may be followed by determining *θ*_*n*,*i*_ and *R*_*n*,*i*_ by means of numerical optimization. Since the above equation will not be solved analytically, it is more convenient to reduce the number of fitting coefficients. This is achieved by testing a number of *s*(*t*) models each obtained by assuming a different value of *n*. Based on Fourier procedure, the larger *n* value considered, the higher model accuracy obtained. In this case, however, a higher accuracy would result with a more complicated model form because of the many periods detected in the data. Accordingly, it can be considered for simplicity *n* = 1. One difficulty remains is that a cosine function crosses a mean equal to zero will produce positive and negative values. In reality, there should be only positive values, while the negative ones should correspond to zero rainfall. To produce zero rainfall, the binary model is used

(4) $$\begin{array}{cc} F\left(t\right) & =\{\begin{array}{cc} s\left(t\right) & \text{if}\;s\left(t\right)\geq 0,\\ 0 & \text{if}\;s\left(t\right)<0. \end{array} \end{array}$$

Following the above procedure, a model of *s*(*t*) is obtained with coefficients shown in Table 1. The overall rainfall model *F*(*t*) is presented in Figure 4(b).

**Figure 7:**
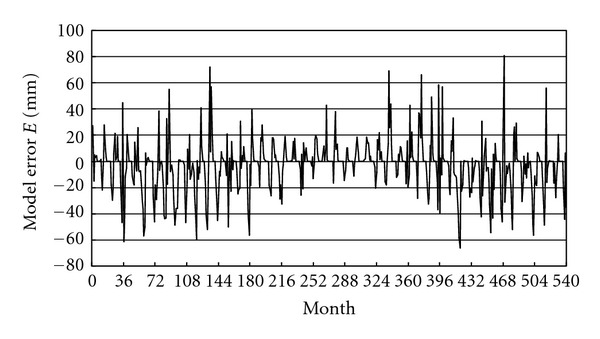
Model error *E* calculated by subtracting the monthly total rainfall data from the calculated values. The data represent that obtained from the weather station of Kuwait Airport for the time duration from January 1965 (corresponding to month number 0) to December 2009 (month number 540).

 Diagnostic checking concerns the verification for the adequacy of the fitted model. The accuracy of the model can be evaluated by calculating the residuals *E* by subtracting the rainfall data from the calculated values. As seen in Figure 7, the model fits the data with some variation representing the remaining stochastic component of time series. The model accuracy can be evaluated quantitatively by estimating the mean absolute error

(5) $$\text{MAE}=\frac{1}{n}\overset{n}{\underset{i=1}{\sum }}\left|E_{i}\right|.$$

It is worth mentioning that this statistical criterion for evaluating model accuracy is more convenient for rainfall data with zero values, while other well-known criteria such as the Nash-Sutcliffe equation and the Mean Absolute Percentage Error are inappropriate, as the actual value in their expressions is found in the denominator by which the equations become undefined for zero rainfalls. It is found that the overall model error estimated by (5) is about MAE = 12.6 mm.

**Figure 8:**
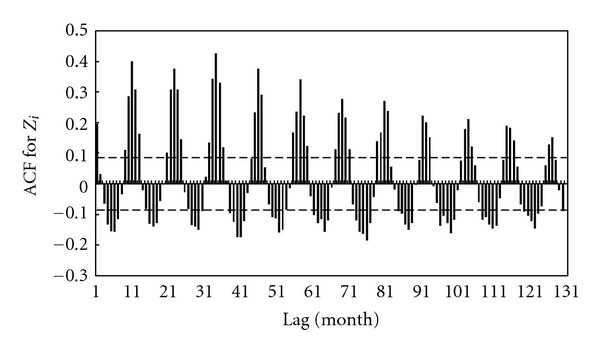
Correlogram up to lag 135 for series *Z*_*i*_. The dashed lines represent upper and lower bounds with significance level *α* = 0.05.

The remaining stochastic component can be tested for any possible persistence and/or random structure. To do this, the residuals are first standardized as follows:

(6) $$Z_{i}=\frac{E_{i}-\bar{E}}{\sigma },$$

where $\bar{E}$ is mean residual of series *E*_*i*_, which is for this data equal to $\bar{E}$ = −5.36 mm; and *σ* is standard deviation of residual of series *E*_*i*_, equal to *σ* = 21.3 mm. The series *Z*_*i*_ then has zero mean and unit standard deviation. The correlogram *r*, also known as the autocorrelation function ACF, can then be obtained for the series *Z*_*i*_ at lag *L* from

(7) $$r_{L}=\frac{\left(1/\left(n-L\right)\right)\sum_{i=1}^{n-L}\left(Z_{i}-\bar{Z}\right)\left(Z_{i+L}+\bar{Z}\right)}{\left(1/n\right)\sum_{i=1}^{n}{\left(Z_{i}-\bar{Z}\right)}^{2}},$$

where $\bar{Z}$ is mean of the sample *n* values of series *Z*_*i*_. The lag *L* is usually taken for values up to *n*/4. The resulting values of *r*_*L*_ will range between −1 and +1.  Figure 8 shows the estimated correlogram. Here, two lines of upper and lower confidence bands were drawn with significance level *α* = 0.05 to examine whether the data are random

(8) $$\text{C}.\text{B}.=\pm \frac{z_{1-\alpha /2}}{\sqrt{n}},$$

where *z* is quantile function of standard normal distribution. If the correlogram is higher (lower) than this upper (lower) band, the null hypothesis that there is no autocorrelation at and beyond a given lag is rejected at the given significance level. As can be seen, the correlogram exhibits an alternating sequence of positive and negative spikes. Such a pattern is the correlogram signature of a repetitive oscillating movement, suggesting that the remaining stochastic component in this data is due to the presence of other periods that have not been taken into account in the model. Nevertheless, owing to the expected complicated behavior of rainfall and to the insufficient data for this station, the remaining stochastic component may be considered nondeterministic.

The role of hidden periods on data modeling can be highlighted by testing the influence of period of 360 months. As it was mentioned earlier, the 360-month period is not present in the periodogram of monthly total rainfall for the station of Kuwait International Airport during the time from January 1965 to December 2009, although it was considered in the process to estimate the model parameters. If this period had been excluded from the model, then the rainfall pattern would have changed as shown in Figure 4(c). Obviously, the model in this case becomes insufficient to capture the large scale of the data pattern. The overall model error here becomes MAE = 13.2 mm, which is about 4.7% larger than before. Accordingly, it can be assumed that the spikes shown in Figure 4(a) such as those for months 35, 135, 339, 376, 396, 470, and 518 that have not been simulated sufficiently by the model may result from hidden periods that had not been taken into account. The implication is that the difficulty in fitting rainfall models by this criterion would require a wide range of historical data sufficient to show all possible periods responsible for the variable pattern.

## 5. Conclusions

This study examined the spatial and temporal variability of monthly total rainfall data obtained from weather stations located in the urban areas of Kuwait. It has been shown that a point estimate of rainfall can be considered spatially representative over the urban areas as the weather stations are sufficiently close in distance. This finding has been proved useful to model rainfall variability over the urban areas using a single dataset obtained from Kuwait International Airport for the time from January 1965 to December 2009. A sinusoidal model has been proposed by considering a number of periods of 6, 12, 18, 20, 26, 30, 42, and 360 months. Except for the last one, all the periods have been identified in the rainfall data of Kuwait International Airport using the periodogram technique. The absence of the last period, which is due to insufficient length of time of data, reveals the role of hidden periods on model accuracy. This is confirmed by plotting the correlogram of the standardized residuals for the remaining stochastic component of the model, which exhibits the signature of a repetitive oscillating movement due to the presence of other periods that have not been taken into account. A wider range of rainfall data would thus be necessary to develop a more accurate periodic model. 

## Figures and Tables

**Table 1:** Estimated coefficients for rainfall model *s*(*t*) with *n* = 1.

*i*	*R*_*n*,*i*_ (mm)	*f*_*i*_ (1/month)	*θ*_*n*,*i*_
1	8	1/6	1
2	30	1/12	0.1
3	7	1/18	−0.2
4	10	1/20	−5
5	4	1/26	−0.9
6	6	1/30	−5.8
7	−6	1/42	3.3
8	−20	1/360	1.3
